# Fragmented QRS and Chagas' disease

**DOI:** 10.1016/s0972-6292(16)30821-x

**Published:** 2014-12-15

**Authors:** Adrian Baranchuk

**Affiliations:** Division of Cardiology, Kingston General Hospital, Queen's University Kingston, Ontario, Canada

**Keywords:** Fragmented QRS, Chagas' disease

I read with interest the manuscript entitled "Repolarization Parameters Are Associated With Mortality In Chagas Disease Patients In The United States" by Bradfield et al., recently published in the Journal [1].

I would like to congratulate the authors for updating information about Chagas' disease (CD) in North America and for advancing concepts on depolarization and repolarization abnormalities in this specific population.

Some aspects of their manuscript though, deserve special attention. The title focuses on repolarization abnormalities, however; fragmented QRS (fQRS) was tested in this population and was significantly different than in the control group [1].

The authors stated "The depolarization parameter, fragmented QRS, is a known marker of increased mortality in CAD and a marker of increased arrhythmic events in CAD, ischemic CM and NICM as well as Brugada syndrome, but has not been previously studied in a CD population. These parameters need further data in CD".

Unfortunately, the authors have missed our 2 publications on this topic which have been tested in a larger population of CD patients with an implantable cardioverter-defibrillator (ICD) (FECHA study) [2,3]. We included 98 patients from 14 centers in Latin America, all of them with CD cardiomyopathy and an implantable cardioverter-defibrillator (ICD). The mean follow-up was 33±20 months. Mean LVEF was 39.6±11.8%. fQRS was found in 56 of the patients (59.6%). Location of the fragmentation was inferior in 57.1%, lateral 35.7% and anterior 44.6%. Rsr pattern was the more prevalent (57.1%) form of fragmentation. Predictors of appropriate therapy in the multivariate model were increased age (p=0.01), secondary prevention indication for the ICD (p=0.01) and ventricular pacing >50% of the time (p=0.004).

The presence of fQRS, in opposition to what was found in patients with CAD and other forms of cardiomyopathy, did not identify patients at higher risk of presenting appropriate therapies delivered by the ICD (p=0.87); regardless of QRS interval duration [4,5].

How can we explain these differences? CD cardiomyopathy is a chronic diffuse myocarditis with patches of fibrosis alternating with healthy myocardial tissue. It is also a dromotropic disorder with frequent involvement of the conduction system. This, even when purely speculative, may help to explain the discrepancies between our studies and Das' publications in populations with CAD.
